# Postpartum Ovarian Vein Thrombophlebitis: A Case Report of Two Patients With Literature Review

**DOI:** 10.7759/cureus.117577

**Published:** 2026-10-07

**Authors:** Hanane Houmaid, Mohamed Amine Guennouni, Abderraouf Soumani

**Affiliations:** 1 Obstetrics and Gynecology Department, Mohammed VI University Hospital Center, Mohammed VI Hospital, Marrakesh, MAR; 2 Obstetrics and Gynecology Department, Mohammed VI University Hospital Center, Marrakesh, MAR; 3 Obstetrics and Gynecology Department, Mohammed VI University Hospital Center, Faculty of Medicine and Pharmacy of Marrakesh, Cadi Ayyad University, Marrakesh, MAR

**Keywords:** computed tomography, low-molecular-weight heparin, maternal mortality, ovarian diseases, puerperal disorders, thrombophlebitis, x-ray

## Abstract

Postpartum ovarian vein thrombophlebitis (OVT) is a rare but potentially life-threatening vascular complication. Its polymorphic clinical presentation and non-specific symptoms frequently lead to diagnostic delays, increasing the risk of thromboembolic complications. We report two cases of postpartum OVT with distinct clinical presentations illustrating the spectrum of disease severity. The first patient was a 23-year-old gravida 3 para 3 with no notable past medical history who presented on postpartum day four with fever and pelvic pain following vaginal delivery. Despite intravenous antibiotic therapy, fever persisted with onset of polyarthralgia. CT angiography revealed extensive thrombosis involving the inferior vena cava, common iliac veins, right external and internal iliac veins, right ovarian vein, and right renal vein. The second patient was a 44-year-old gravida 4 para 3 with severe rheumatic valvular disease and pulmonary hypertension on antiplatelet therapy who underwent cesarean delivery for fetal distress. On postpartum day two, she developed diffuse abdominal pain with guarding and fever, with C-reactive protein elevation from 23 to 259 mg/L. Abdominopelvic CT demonstrated bilateral ovarian vein thrombosis (right: 16 mm, left: 12 mm). Antiplatelet therapy was discontinued, and both patients were treated with therapeutic-dose low-molecular-weight heparin and broad-spectrum antibiotics, and improved clinically. These cases highlight the diagnostic challenges of postpartum OVT, particularly its variable clinical presentation and spectrum of severity. Persistent postpartum fever unresponsive to antibiotics should raise suspicion of OVT and prompt early imaging; contrast-enhanced CT was the modality used in both cases. Both patients improved clinically under combined anticoagulant and antibiotic therapy, although no conclusion on efficacy can be drawn from two cases, and the shorter anticoagulation duration in Case 2 was an individualized decision that should not be generalized. Multidisciplinary involvement was important in our complex case.

## Introduction

Postpartum ovarian vein thrombophlebitis (OVT) is a rare form of puerperal venous thromboembolism in which a clot forms within one or both ovarian veins, the vessels that drain blood from the ovaries into the pelvic venous system. In this report, “ovarian vein thrombosis” and “OVT” are used interchangeably, while “thrombophlebitis” is used when the thrombosis is associated with an infectious or inflammatory process, as in our patients. The condition affects fewer than 1 in 500 deliveries, with a higher frequency after cesarean section, and it usually becomes apparent during the first week after delivery.

Although uncommon, OVT can extend to the iliac veins, the inferior vena cava, and the renal veins, and may be complicated by pulmonary embolism or sepsis. Its diagnosis is challenging because fever and pelvic or abdominal pain, its most frequent presenting features, overlap considerably with far more common postpartum conditions such as endometritis, urinary tract infection, and appendicitis. No single symptom, sign, or laboratory test is specific enough to confirm the diagnosis; persistent fever despite appropriate antibiotic therapy should therefore prompt consideration of OVT and early imaging. Once the diagnosis is established, management combines anticoagulation with antibiotic therapy when an infection is suspected, and the outcome is usually favorable when treatment is started early.

Despite growing recognition of this condition, most available reports are isolated cases or heterogeneous series, and data on atypical presentations and on complex clinical contexts remain limited. In particular, extensive thrombotic extension after vaginal delivery in a patient without comorbidities, bilateral involvement, OVT in women with severe cardiac disease and pulmonary hypertension, and the choice of anticoagulation duration in provoked cases at high hemorrhagic risk are insufficiently documented. We report two cases managed in our department that illustrate these situations: an indolent but extensive thrombosis extending to the inferior vena cava, iliac veins, and right renal vein after vaginal delivery, and a bilateral OVT after cesarean delivery in a patient with severe rheumatic valvular disease and pulmonary hypertension on antiplatelet therapy. The objective of this report is to compare these contrasting presentations, to discuss their diagnostic and therapeutic management, including an individualized anticoagulation strategy, and to highlight the importance of early imaging in persistent postpartum fever unresponsive to antibiotics.

## Case presentation

Case 1

A 23-year-old gravida 3 para 3 with no notable past medical history presented on postpartum day four following vaginal delivery with fever and pelvic pain. Initial examination revealed a febrile patient with malodorous lochia and pain on uterine mobilization. The patient’s clinical course under intravenous antibiotic therapy was marked by persistent fever, onset of polyarthralgia, and resolution of pelvic pain. Vaginal culture revealed a multisensitive *Escherichia coli* infection. Abdominopelvic CT angiography established the diagnosis of deep pelvic thrombophlebitis with extensive involvement of the inferior vena cava, common iliac veins, right external and internal iliac veins, right ovarian vein, and right renal vein (Figure 1). A thrombophilia workup performed before initiating anticoagulation returned normal results. The patient was started on therapeutic-dose low-molecular-weight heparin (enoxaparin 1 mg/kg twice daily) combined with broad-spectrum antibiotic therapy consisting of intravenous ceftriaxone 2 g/day and intravenous metronidazole 500 mg three times daily, consistent with the identified multisensitive *E. coli* infection and the polymicrobial risk associated with postpartum pelvic sepsis. Following clinical improvement, intravenous therapy was relayed to oral formulations, with a total antibiotic duration exceeding 14 days. The clinical course improved progressively under combined treatment, with anticoagulation continued for a total of three months.

**Figure 1:**
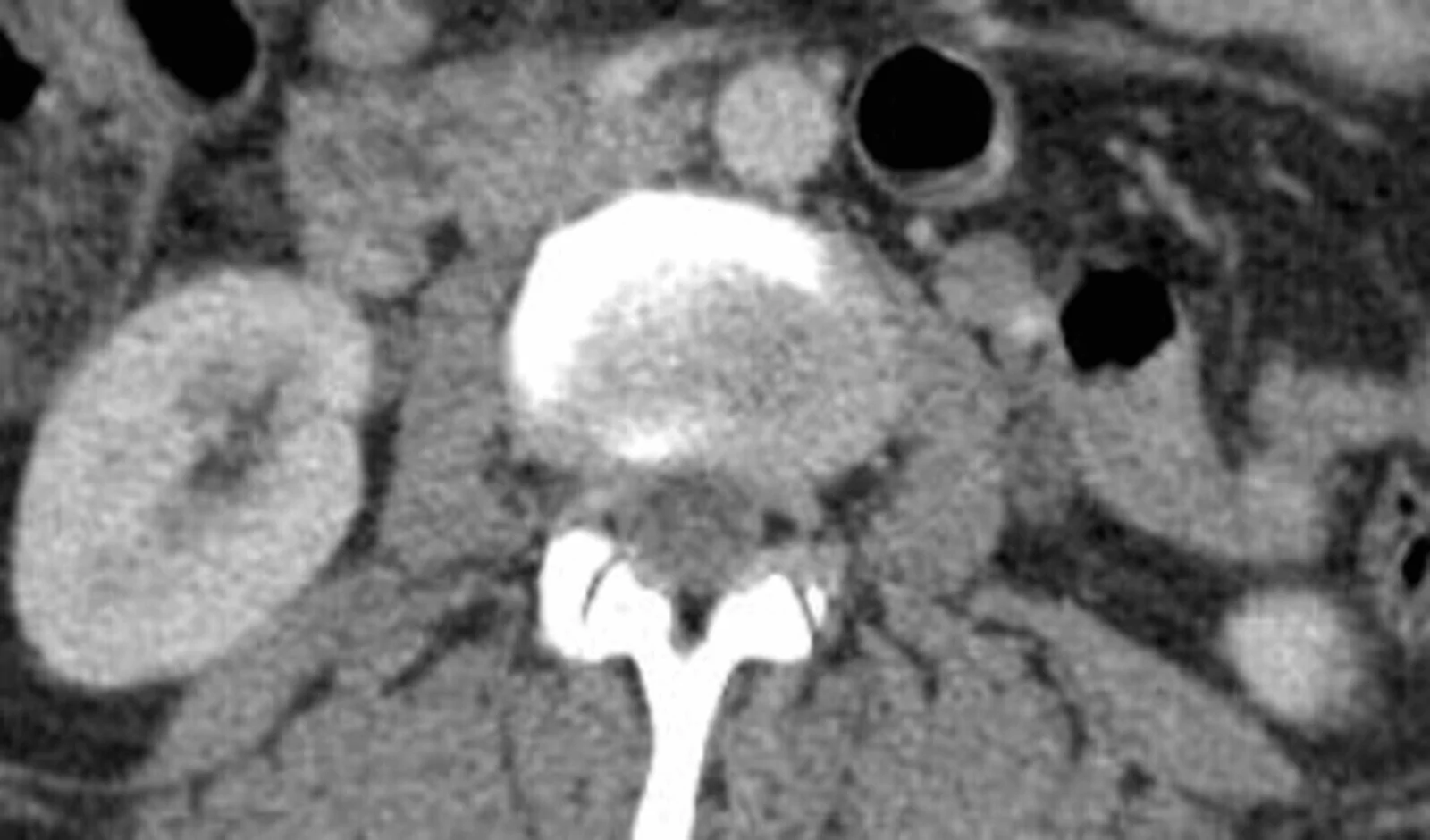
Abdominopelvic CT angiography revealing extensive ovarian vein thrombosis with caval extension. CT images demonstrating thrombosis involving the right ovarian vein with extension to the inferior vena cava, common iliac veins, right external and internal iliac veins, and right renal vein (Case 1).

Case 2

A 44-year-old gravida 4 para 3 who had previously delivered three living children vaginally was followed for severe rheumatic valvular disease complicated by pulmonary hypertension, with preserved left ventricular systolic function, and had been placed on antiplatelet-dose acetylsalicylic acid during pregnancy. Clinical examination at admission revealed a conscious, normotensive patient with normal heart rate and no signs of dyspnea. Obstetric examination was unremarkable. The patient was admitted not in active labor at 37 weeks of gestation, and obstetric ultrasound showed a viable singleton pregnancy in cephalic presentation, normal amniotic fluid volume, fundal placenta, and an estimated fetal weight of 2,700 g. Laboratory workup at admission revealed isolated thrombocytopenia at 106,000/µL, which therefore predated both delivery and the thrombotic event. On postpartum day two, the C-reactive protein (CRP) had risen from 23 mg/L to 259 mg/L, with leukocytosis (white blood cell count: 15,000/µL) and lymphopenia (730/µL).

The patient underwent labor induction under epidural anesthesia with misoprostol, provided fetal heart rate monitoring was reassuring. After six hours of labor, monitoring revealed multiple decelerations reaching 90 beats/minute unresponsive to intrauterine resuscitation measures, with the patient in early labor with cervical dilation at 2 cm. The patient was transferred to the operating room due to non-reassuring fetal status and delivered a female newborn by cesarean section with an Apgar score of 10/10 and birth weight of 2,650 g.

During postoperative surveillance, on postpartum day two, the patient developed diffuse abdominal pain with diffuse guarding in a febrile context. Laboratory workup revealed hyperneutrophilia at 15,000/µL, lymphopenia at 730/µL, and CRP elevation from 23 to 259 mg/L. Abdominopelvic CT demonstrated dilation of the ovarian veins measuring 16 mm on the right and 12 mm on the left, with rim enhancement and intraluminal material that was spontaneously hypodense and non-enhancing after contrast injection, consistent with bilateral ovarian vein thrombosis (Figure 2). Aspirin was discontinued, and the patient was started on enoxaparin 60 mg (6,000 international units) twice daily along with antibiotic therapy with ceftriaxone 2 g daily. A thrombophilia workup was not performed in this patient, given that the thrombotic event was considered provoked in the context of cesarean delivery, severe underlying cardiac disease with pulmonary hypertension, and perioperative hemodynamic changes. These comorbidities were deemed sufficient to explain the bilateral thrombotic involvement without requiring further thrombophilia investigation at this stage. CRP was monitored every 48 hours until normalization on day eight. The patient’s clinical course improved progressively. Anticoagulation was continued for a total of six weeks, a duration deemed appropriate given that the thrombotic event was fully provoked, occurring in the immediate perioperative setting of cesarean delivery, and in view of the patient’s severe underlying cardiac disease requiring close cardiological monitoring and individualized antithrombotic management. Although current evidence, including the scoping review by Monnet et al. [1], recommends a minimum of three months for symptomatic postpartum OVT, this duration is shorter than the minimum of three months recommended in current reviews [1]. It reflects an individualized decision in a single patient with a high-risk cardiac condition, based on clinical and biological resolution (CRP normalization on day eight). In the absence of follow-up imaging or long-term follow-up, the safety of this shorter course cannot be established from this case and should not be generalized. In this patient, CRP normalization at day eight and progressive clinical improvement supported this individualized approach. Laboratory findings for both patients are summarized in Table 1. Clinical characteristics of both patients are summarized in Table 2.

**Figure 2:**
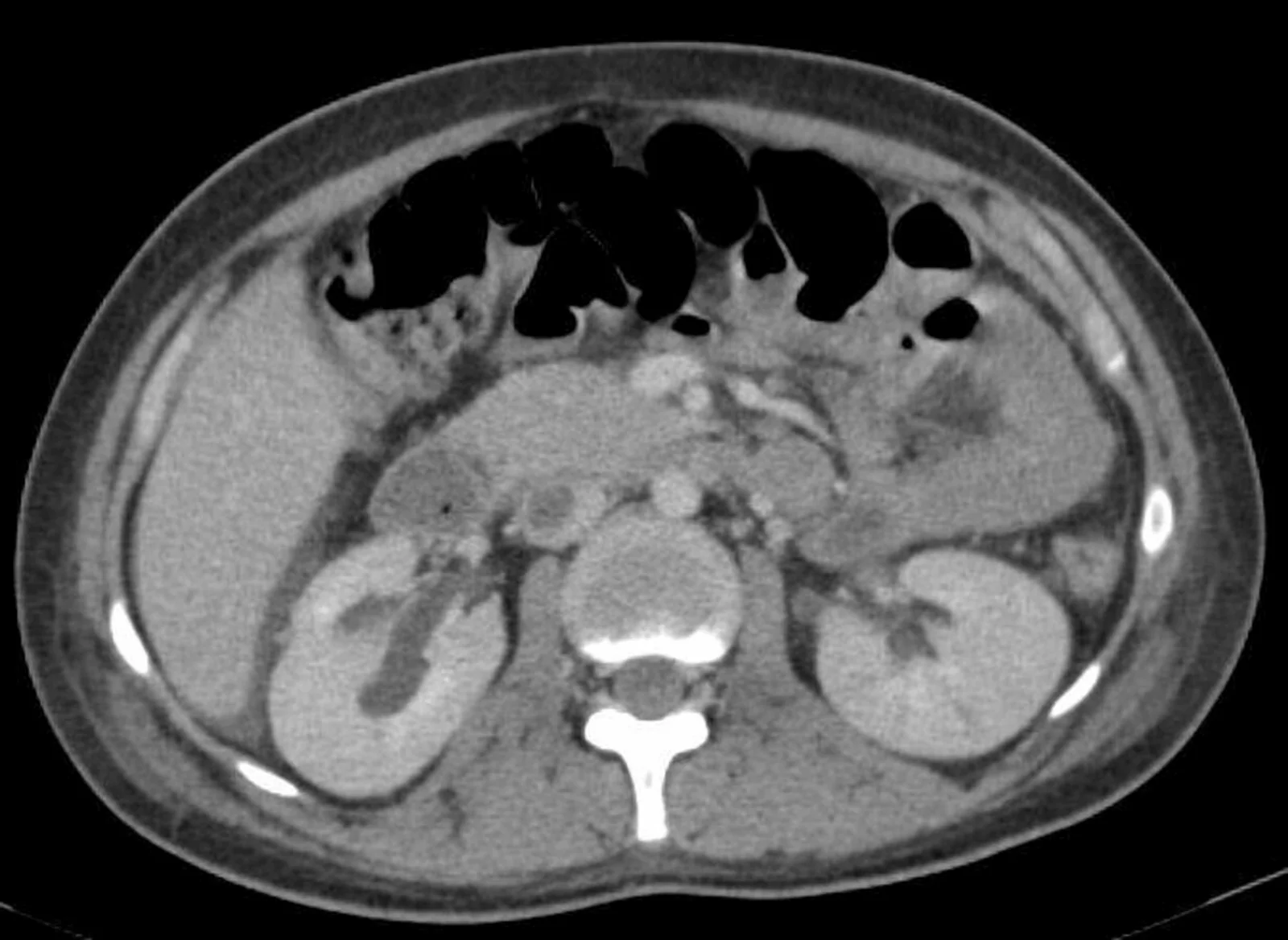
Contrast-enhanced pelvic CT demonstrating bilateral ovarian vein thrombophlebitis. Axial CT images showing dilated ovarian veins (right: 16 mm, left: 12 mm) with rim enhancement and intraluminal hypodense non-enhancing material consistent with bilateral thrombosis (Case 2).

**Table 1:** Laboratory findings. Not available: values not retrievable from the retrospective records.

Parameter	Case 1	Case 2	Reference Range	Interpretation
Microbiology	Vaginal culture: *E. coli* (multisensitive)	Not performed	—	Genital tract infection confirmed (Case 1)
C-reactive protein	Not available	23 mg/L → 259 mg/L (by postpartum day 2); normalized on day 8	<5 mg/L	Marked inflammatory response (Case 2)
White blood cell count	Not available	15,000/µL (postpartum day 2)	4,000–10,000/µL	Leukocytosis (Case 2)
Lymphocyte count	Not available	730/µL (postpartum day 2)	1,000–4,000/µL	Lymphopenia (Case 2)
Platelet count	Not available	106,000/µL (at admission, before delivery)	150,000–450,000/µL	Pre-existing thrombocytopenia (Case 2)
Thrombophilia workup	Normal (before anticoagulation)	Not performed	—	See Discussion for rationale

**Table 2:** Summary of the clinical characteristics of the two patients.

Variable	Case 1	Case 2
Age (years)	23	44
Gravidity/Parity	G3P3	G4P3
Comorbidities	None	Severe rheumatic valvular disease, pulmonary hypertension (on aspirin)
Mode of delivery	Vaginal	Cesarean (fetal distress)
Postpartum day of presentation	Day 4	Day 2
Main symptoms	Fever, pelvic pain, malodorous lochia; later polyarthralgia	Fever, diffuse abdominal pain, guarding
Laterality	Right	Bilateral (right: 16 mm, left: 12 mm)
Extent of thrombosis	Ovarian vein, inferior vena cava, common/external/internal iliac veins, right renal vein	Ovarian veins only
Imaging	CT angiography	Contrast-enhanced CT
Anticoagulation	Enoxaparin 1 mg/kg twice daily, 3 months	Enoxaparin 60 mg twice daily, 6 weeks
Antibiotics	Ceftriaxone + metronidazole, >14 days	Ceftriaxone
Outcome	Progressive clinical improvement	Progressive clinical improvement; C-reactive protein normalized on day 8

## Discussion

Epidemiology and risk factors

Postpartum OVT is a rare but potentially life-threatening condition. According to the most recent scoping review by Monnet et al. (2024) [1], which pooled 19 studies including 1,128 patients, its frequency in the postpartum setting is 0.06% of deliveries overall, rising to 0.19% after cesarean section. Persistent fever despite antibiotic therapy was associated with OVT in up to 23% of postpartum cases in the same review. The right ovarian vein is affected in 70-90% of cases, owing to its greater length, incompetent valves, acute angle of entry into the inferior vena cava, and the physiological dextrorotation of the gravid uterus [2]. Well-established risk factors include cesarean section, postpartum endometritis, and a prior history of venous thromboembolism [3]. A retrospective French study by Lebon et al. [4] confirmed endometritis and cesarean delivery as the two main independent risk factors for postpartum OVT. Assisted reproductive technology and multiple pregnancies have also been identified as emerging risk factors [2]. The second case in our series illustrates an underreported additional risk factor: severe pre-existing cardiac disease associated with pulmonary hypertension, which, combined with cesarean delivery, likely contributed to the bilateral thrombotic involvement, a presentation reported in only 6-14% of cases in the literature [2].

Pathophysiology: Virchow’s triad revisited

The pathophysiology of OVT is explained by Virchow’s triad, whose three components converge during the postpartum period to create an ideal thrombogenic environment [5]. Hypercoagulability results from physiological prothrombotic changes of pregnancy that persist for up to six weeks postpartum: increases in coagulation factors II, VII, VIII, X, and von Willebrand factor, combined with a decrease in protein S levels and resistance to activated protein C [5,6]. Venous stasis is promoted by marked dilation of the ovarian veins during pregnancy (diameter increasing up to three-fold, with a 60-fold increase in flow capacity) followed by an abrupt postpartum decrease in flow velocity, particularly in the right ovarian vein where antegrade flow coexists with countercurrent flow in the left [2]. Endothelial injury may result from direct surgical trauma during cesarean section, local spread of bacterial infection from the uterus to the venous wall, or systemic inflammatory insult mediated by pro-inflammatory cytokines [5]. Anatomical factors specific to the right ovarian vein, its longer and more tortuous course, its acute angle of drainage into the inferior vena cava, and its weaker valvular apparatus, explain the well-established right-sided predominance of the disease [2].

Clinical aspects: A polymorphic presentation

The diagnosis of OVT remains a major clinical challenge due to its protean and non-specific presentation. According to Riva and Calleja-Agius’s narrative review (2021) [3], OVT most commonly manifests two to six days after delivery. Cardinal symptoms include abdominal pain with right-sided predominance, persistent fever, and occasionally a palpable adnexal mass (reported in only 46% of cases). Fever resistant to antibiotic therapy is the most consistent clinical trigger for suspecting OVT: in the scoping review by Monnet et al. (2024) [1], persistent fever after three to five days of antibiotic treatment was identified as the main clinical indicator leading to the diagnosis. In Wang et al.’s case report (2023) [7], OVT was diagnosed after vaginal delivery based on fever and left lower abdominal pain appearing within 24 hours, demonstrating that the condition is not limited to the right side or to cesarean section. Both patients in our series presented with persistent fever unresponsive to antibiotics, which prompted imaging and led to diagnosis.

Diagnostic approach

Laboratory findings are non-specific, typically showing elevated CRP and neutrophilic leukocytosis; blood cultures are positive in fewer than half of cases, most commonly isolating *E. coli* or Gram-positive organisms [3]. The imaging strategy is central to diagnosis. Doppler ultrasound remains the initial first-line examination owing to its wide availability, low cost, and absence of radiation; however, its sensitivity for OVT is limited (approximately 52% for transabdominal approach) due to operator dependence and interference from bowel gas [8]. CT with contrast injection is the most commonly used modality in clinical practice. El-Diasty and Noorelahi (2023) [8] confirmed that contrast-enhanced CT allows visualization of the intraluminal filling defect, peripheral rim enhancement, and extension of the thrombus to adjacent vessels, making it the most commonly used modality for confirming the diagnosis in clinical practice. The venous phase should ideally be acquired 60-80 seconds after contrast injection for optimal opacification of ovarian veins. MRI offers the highest diagnostic accuracy: in the scoping review by Monnet et al. [1], MRI was associated with the best overall diagnostic performance, with sensitivity and specificity reaching 92-100% and 100%, respectively, and is particularly valuable for assessing the superior extent of a thrombus involving the inferior vena cava or renal veins, and for patients in whom iodinated contrast is contraindicated [9]. Both cases in our series were diagnosed by contrast-enhanced CT angiography, which confirmed venous dilation, endoluminal thrombus with rim enhancement, and, in Case 1, extensive caval and renal vein involvement.

Differential diagnoses

The broad differential diagnosis of OVT includes postpartum endometritis, pyelonephritis, appendicitis, pelvic abscess, adnexal torsion, and broad ligament hematoma. In the series by Akbari et al. (2023) [10], reviewing septic pelvic thrombophlebitis at a tertiary center, OVT was confirmed only after the failure of antibiotic therapy in all cases, highlighting how frequently the diagnosis is delayed. Tsitlakidis et al. (2021) [11] reported a case initially managed as acute appendicitis before CT revealed right OVT with renal extension, illustrating the diagnostic pitfall. In clinical practice, we suggest that persistence of fever beyond 48-72 hours under well-conducted antibiotic therapy should systematically prompt consideration of OVT and initiation of dedicated imaging.

Ovarian vein thrombosis and septic pelvic thrombophlebitis are overlapping rather than mutually exclusive entities. Thrombosis of the ovarian vein defines the vascular lesion, whereas the term thrombophlebitis implies an infected or inflammatory thrombus. In Case 1, the clinical picture (fever, malodorous lochia, uterine tenderness) and the multisensitive *E. coli* isolated from the vaginal culture were consistent with puerperal genital tract infection, and persistent fever despite antibiotics suggested secondary involvement of the pelvic veins. However, a vaginal culture documents infection of the genital tract and does not by itself prove that the thrombus is infected. In Case 2, no microbiological sample was obtained, and the septic component was inferred from the marked inflammatory syndrome and the response to combined anticoagulant and antibiotic therapy. We therefore consider both cases as ovarian vein thrombosis with a probable infectious component.

Therapeutic management

Therapeutic management combines anticoagulation and antibiotic therapy. Low-molecular-weight heparin at therapeutic doses remains the anticoagulant of choice, particularly in the postpartum and breastfeeding context, as confirmed by the most recent scoping review by Monnet et al. (2024) [1] and the systematic review and meta-analysis by Riva et al. (2024) [12]. The latter, a meta-analysis that pooled 17 observational studies comprising 621 anticoagulated OVT patients, reported a low rate of major bleeding (1.27%) and of recurrent venous thromboembolism [12]. However, these data come from observational studies and do not allow causal conclusions on the efficacy or safety of anticoagulation. The optimal duration of anticoagulation remains debated. Most authors recommend three to six months for symptomatic postpartum OVT [1,3,12]. The recent review by Monnet et al. [1] concluded that a minimum of three months is appropriate for provoked OVT (postpartum, surgical), extending to six months when significant risk factors are present. Transition to direct oral anticoagulants (DOACs) after the postpartum or breastfeeding period is emerging as a viable option: the DUST registry (ongoing) specifically addresses DOACs in unusual-site venous thromboembolism, including OVT, but robust prospective data are still lacking in this specific population [12]. Broad-spectrum antibiotic therapy targeting Gram-negative organisms and anaerobes is indicated whenever associated infection is suspected [3]. Surgical interventions (venous ligation, thrombectomy) are reserved for refractory cases and carry significant morbidity [1].

Both patients presented within the typical window of two to six days after delivery described in the literature [3]. Bilateral involvement, present in one of our two patients, is reported in only 6-14% of published cases [2]. With two patients, no frequency can be inferred, but this case suggests that severe cardiac disease with pulmonary hypertension may be an additional context for bilateral thrombosis. Our series adds three observations: extensive caval and renal vein extension can occur after vaginal delivery in a young patient without comorbidities; bilateral OVT can occur in the setting of severe cardiac disease; and anticoagulation duration may need to be individualized in high-risk provoked cases.

Complications and prognosis

The most feared complications are pulmonary embolism, thrombotic extension to the inferior vena cava and renal veins, and septic shock. In the scoping review by Monnet et al. [1], pulmonary embolism occurred in 6.5% of patients, while extension to the iliac veins, inferior vena cava, and left renal vein occurred in 5.9%, 10.3%, and 9.6% of cases, respectively. Murata et al. (2022) [13] reported an asymptomatic postpartum OVT extending toward the inferior vena cava with a potential risk of pulmonary thromboembolism, highlighting the importance of vigilance even in the absence of classic symptoms. Cheung et al. (2024) [14] reported a severe case of right OVT with left renal venous infarction after vaginal delivery, illustrating the risk of contiguous extension. In our patients, when treatment was initiated promptly, the clinical course typically improved within 48-72 hours. In a meta-analysis of observational studies [12], overall mortality in treated patients was below 2.5%, and recurrent venous thromboembolism is rare during anticoagulation. Recanalization on follow-up imaging occurs in approximately 70% of patients [1]. The management and clinical characteristics of OVT across diverse populations have been further detailed in a retrospective case series by Alsheef et al. (2022) [15].

Prevention

Systematic screening for OVT is not recommended in asymptomatic postpartum patients. However, thrombophilia workup is warranted in idiopathic OVT or when a first-degree family history of venous thromboembolism is identified, as thrombophilia was detected in 18% of tested patients in the scoping review by Monnet et al. [1], and its presence may guide the duration of anticoagulation and inform prophylactic strategies in subsequent pregnancies. Preventive measures include early postoperative mobilization, adequate hydration, and thromboprophylaxis with low-molecular-weight heparin after cesarean section in at-risk patients, in accordance with current obstetric venous thromboembolism guidelines [6]. Rigorous perioperative antibiotic prophylaxis also contributes to reducing the risk of infectious thrombophlebitis.

Limitations

This report has several limitations: its retrospective design, incomplete laboratory data in Case 1, the absence of microbiological confirmation of thrombus infection, the absence of patient body weights in the available records, and the absence of follow-up imaging.

## Conclusions

Postpartum OVT, although rare, can be a serious obstetric complication. In our two patients, persistent fever with abdominal or pelvic pain despite antibiotic therapy prompted imaging, which led to the diagnosis; therefore, we suggest that OVT be considered in this situation. Contrast-enhanced CT was the modality used in both cases and is the most commonly used in the literature, although the choice of imaging should be adapted to availability and clinical circumstances (Doppler ultrasound as an initial examination, MRI when iodinated contrast is contraindicated or to assess the upper extent of the thrombus). Both patients improved clinically under therapeutic-dose low-molecular-weight heparin and antibiotics, but no conclusion on efficacy can be drawn from two cases. The six-week anticoagulation course in Case 2 was an individualized decision in a high-risk patient; it differs from the minimum of three months reported in the literature and should not be considered a recommendation. The optimal duration of anticoagulation remains debated, and prospective studies are needed to optimize therapeutic and preventive strategies, particularly regarding DOACs. Multidisciplinary management, including cardiology, was important in our complex case.

## References

[1] Monnet M, Dufrost V, Wahl D, Morel O, Agopiantz M, Zuily S (2024). Epidemiology, natural history, diagnosis, and management of ovarian vein thrombosis: a scoping review. J Thromb Haemost.

[2] Huynh QH, Pham HD, Truong QO, Luong MT, Ho TT, Ho XT (2025). Postpartum ovarian vein thrombosis: report of 2 cases and review of diagnosis. Radiol Case Rep.

[3] Riva N, Calleja-Agius J (2021). Ovarian vein thrombosis: a narrative review. Hamostaseologie.

[4] Lebon A, Connault J, Cardaillac C, Thubert T, Winer N, Dochez V (2022). [Postpartum ovarian vein thrombophlebitis: diagnosis, treatment and follow-up. Retrospective study over 10 years]. Rev Med Interne.

[5] Ferguson SM, Arbona D, Furiato A (2022). Postpartum ovarian vein thrombosis: case report. Clin Pract Cases Emerg Med.

[6] Maughan BC, Marin M, Han J (2022). Venous thromboembolism during pregnancy and the postpartum period: risk factors, diagnostic testing, and treatment. Obstet Gynecol Surv.

[7] Wang JJ, Hui CC, Ji YD, Xu W (2023). Computed tomography diagnosed left ovarian venous thrombophlebitis after vaginal delivery: a case report. World J Clin Cases.

[8] El-Diasty MT, Noorelahi Y (2023). Imaging findings of ovarian vein thrombosis. Cureus.

[9] Bannow BT, Skeith L (2017). Diagnosis and management of postpartum ovarian vein thrombosis. Hematology Am Soc Hematol Educ Program.

[10] Akbari EA, Majdalawi R, Harb DK, Hazari K, Abdelkareem W, Ammar A (2023). Postpartum septic pelvic thrombophlebitis in a tertiary maternity hospital in Dubai, UAE. Cureus.

[11] Tsitlakidis C, Al Ajmi KI, Al Madhani AY, Ahmidat AH (2021). Postpartum ovarian vein thrombosis manifesting as acute appendicitis: a case report. J Med Case Rep.

[12] Riva N, Muscat-Baron L, Vassallo C (2024). Safety and efficacy of anticoagulant treatment in patients with ovarian vein thrombosis: a systematic review and meta-analysis of observational studies. Res Pract Thromb Haemost.

[13] Murata T, Yoshimoto Y, Shibano Y, Owada K, Miyajima M, Nakamura S, Yamauchi R (2022). Potential critical risks of pulmonary thromboembolism from an asymptomatic postpartum ovarian vein thrombosis: a case report. BMC Pregnancy Childbirth.

[14] Cheung W, Stegwee S, van Hamont D, Visser J (2024). Postpartum ovarian vein thrombosis with left renal venous infarction after vaginal delivery. BMJ Case Rep.

[15] Alsheef M, Abuzied Y, Alosaimi M (2022). Clinical characteristics and management of ovarian vein thrombosis: a case series. Front Cardiovasc Med.

